# Financial toxicity and acute injury in the Kilimanjaro region: An application of the Three Delays Model

**DOI:** 10.1371/journal.pone.0308539

**Published:** 2024-08-30

**Authors:** Parker Frankiewicz, Yvonne Sawe, Francis Sakita, Blandina T. Mmbaga, Catherine Staton, Anjni P. Joiner, Emily R. Smith

**Affiliations:** 1 Global Emergency Medicine Innovation and Implementation Research Center, Duke Global Health Institute, Durham, NC, United States of America; 2 Kilimanjaro Christian Medical Centre, Moshi, Tanzania; 3 Kilimanjaro Christian Medical University College, Moshi, Tanzania; 4 Kilimanjaro Clinical Research Institute, Moshi, Tanzania; 5 Department of Emergency Medicine, Duke University School of Medicine, Durham, NC, United States of America; 6 Department of Surgery, Duke University School of Medicine, Durham, NC, United States of America; Imperial College London School of Public Health, UNITED KINGDOM OF GREAT BRITAIN AND NORTHERN IRELAND

## Abstract

**Background:**

Trauma and injury present a significant global burden–one that is exacerbated in low- and middle-income settings like Tanzania. Our study aimed to describe the landscape of acute injury care and financial toxicity in the Kilimanjaro region by leveraging the Three Delays Model.

**Methods:**

This cross-sectional study used an ongoing injury registry and financial questionnaires collected at Kilimanjaro Christian Medical Centre (KCMC) in Moshi, Tanzania from December 2022 until March 2023. Financial toxicity measures included catastrophic expenditure and impoverishment, in accordance with World Health Organization standards. Descriptive analysis was also performed.

**Findings:**

Most acute injury patients that presented to the KCMC Emergency Department experienced financial toxicity due to their out-of-pocket (OOP) hospital expenses (catastrophic health expenditure, CHE: 62.8%; impoverishment, IMP: 85.9%). Households within our same which experienced financial toxicity had more dependents (CHE: 18.4%; IMP: 17.9% with ≥6 dependents) and lower median monthly adult-equivalent incomes (CHE: 2.53 times smaller than non-CHE; IMP: 4.27 times smaller than non-IMP). Individuals experiencing financial toxicity also underwent more facility transfers with a higher surgical burden.

**Interpretation:**

Delay 1 (decision to seek care) and Delay 2 (reaching appropriate care facility) could be significant factors for those who will experience financial toxicity. In the Tanzanian healthcare system where national health insurance is present, systematic expansions are indicated to target those who are at higher risk for financial toxicity including those who live in rural areas, experience unemployment, and have many dependents.

## Introduction

Trauma and injuries account for more than 5 million deaths per year. This amounts to more annual deaths on a global scale than tuberculosis, malaria, and HIV/AIDS combined with 90% of those deaths concentrated in low- and middle-income countries (LMICs) [1]. Nearly a billion more individuals sustain non-fatal injuries requiring medical care, inflating disability-adjusted life years (DALYS) in LMICs to 1.4 times greater than the global average [2–4]. Post-injury prognosis is also worse in LMICs often due to late hospital presentation and scarce diagnostic imaging, amongst other delays to care [2]. If these disparities were reduced, around one-third of the global traumatic injury deaths could be avoided [3, 4]. In many of these LMIC settings, financial toxicity has been found to be a strong predictor of delays in a patient’s healthcare pathway, contributing to poorer prognoses [5–8].

Financial toxicity can be assessed in a variety of ways, but two main indicators leveraged by the World Health Organization (WHO) are catastrophic health expenditure and impoverishment. A catastrophic health expenditure (CHE) is defined as an out-of-pocket (OOP) payment that exceeds a set proportion of total household expenditure at which point that household must sacrifice basic needs, sell assets, and/or take on debts [9]. Impoverishment (IMP) occurs when a household is pushed below the poverty line by OOP costs [10]. As a result, the United Nations (UN) have prioritized reductions in CHE and IMP incidence to expand universal health coverage and human development in developing healthcare systems [11, 12]. Universal health coverage (UHC) is touted by the WHO and UN as a prominent strategy for equitable access to healthcare services through reductions in financial toxicity [10, 13]. Despite the international priority and utility of financial toxicity, there is a paucity of studies focusing on financial toxicity within the context of traumatic injuries in LMICs.

Moshi is a city that lies at the foot of Mount Kilimanjaro within Tanzania, which is an LMIC per the World Bank’s Atlas method [14]. The Kilimanjaro region is fraught with intense road traffic with minimal government oversight—resulting in rising injury rates which stem from a steady increase in road traffic collisions, amongst other sources [15]. Previous studies at one of the largest zonal referral hospitals in the Kilimanjaro region, Kilimanjaro Christian Medical Center (KCMC), have shown that improper recognition of injury severity in prehospital and hospital transfer systems might contribute to delays in care and poor outcomes [16]. However, there is a lack of in-depth investigation regarding the financial factors which could influence how patients suffering from traumatic injuries access the medical system, if at all. We conducted a cross-sectional study of acute injury patients to investigate financial toxicity alongside other barriers and facilitators to emergency care in the Kilimanjaro region of Tanzania. Recognizing that delays to care can drive patient outcomes and financial toxicity, we leveraged the Three Delays Model as a framework for our results [17]. Our study engaged patients in KCMC’s Emergency Department (ED) to lay the foundation for future solutions that facilitate access to emergency care in the wake of a traumatic injury.

## Methods

### Overall framework

**Fig 1:**
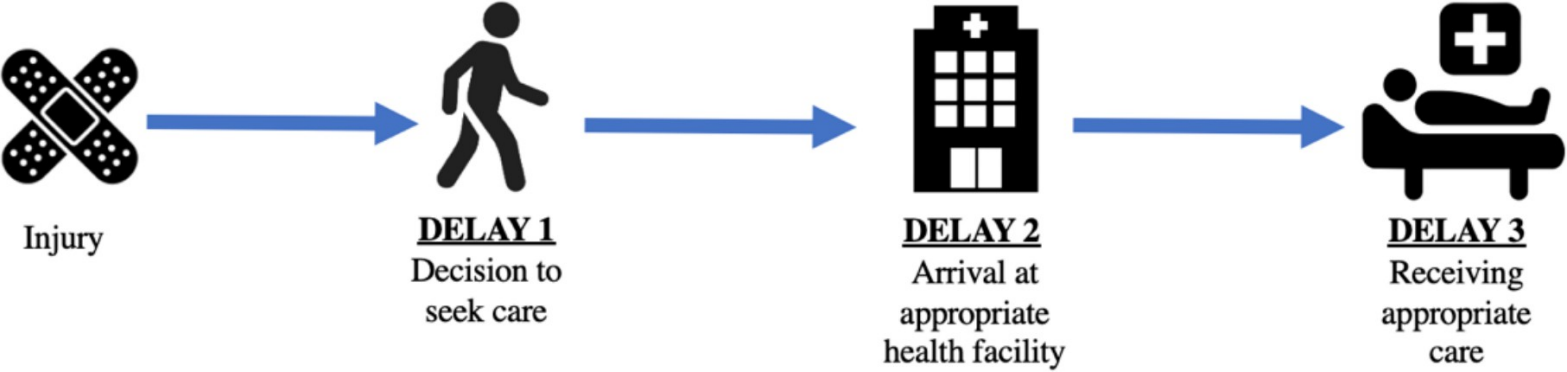
The Three Delays Model, revisualized for acute injury.

There is evidence and precedent that supports leveraging a pre-existing framework to better understand the landscape of emergency care in the Kilimanjaro region, such as the Three Delays Model [18, 19]. While originally designed for maternal mortality in LMICs, the Three Delays model is equally applicable to traumatic injuries due to the shared time-critical nature of the two dispositions [17, 19, 20]. These delays in an individual’s path to reach definitive care are categorized in three steps– 1) delays in the decision to seek care; 2) delays in reaching care; and 3) delays in receiving care (Fig 1) [17]. Each of the three delays can be exacerbated or improved by numerous factors. The first delay can be impacted by sociocultural influences on patient agency, a perceived injury severity, and potential OOP costs even when insured [5, 21, 22]. Distance to a healthcare facility or potential costs of transportation can influence the second delay [21, 22]. When staff or equipment are in short supply or there is inequitable patient prioritization, patients can suffer from the third delay [21, 22]. Developing a better understanding of a patient’s prehospital care path could facilitate the seeking, reaching, and receiving of emergency care in an appropriate, timely, and safe manner—ultimately improving patient outcomes [23].

### Setting

KCMC is a zonal referral hospital located in Moshi, Tanzania with a catchment area encompassing the Kilimanjaro region, serving an estimated 15 million people. KCMC’s ED sees approximately 24,000 patients annually, with around 2,000 of those patients reporting an injury. The Tanzanian health system is a semi-rigid system based upon designated referral pathways. A patient who is dependent on the Tanzanian National Health Insurance Fund (NHIF) and cannot afford healthcare expenses out-of-pocket must first seek care at a local facility prior to receiving a referral for care at a more advanced facility, regardless of complaint. Since 2012, a local registry of injury patients has collected demographics, injury data, and other patient characteristics at KCMC’s ED.

### Participant enrollment

Every patient that presented to KCMC’s ED within the study timeframe was screened for eligibility and approached for potential enrollment if eligible. To be included in the study, patients had to be ≥18 years old, seeking care for an acute injury (presenting to the ED ≤24 hours after injury), and able to speak with research personnel. Patients deemed incapacitated upon arrival by the treating physician who had a Legal Authorized Representative (LAR) provide informed consent were also included and then re-consented upon regaining their ability to consent. Exclusion criteria included patient or family refusal, patients complaining of an injury ≥24 hours old, incapacitated patients without a LAR, and patients ≤18 years old.

### Ethics statement

Formal, informed consent was obtained in written format from either the participant or their LAR. Ethics approval obtained from Duke University IRB, KCMC IRB, and Tanzania’s National Institute for Medical Research (NIMR).

### Data collection

This was a cross-sectional study that utilized data from two main sources: a prospective trauma registry and a combined health needs and financial status assessment. Both were collected at the Kilimanjaro Christian Medical Centre in Moshi, Tanzania between December 1st, 2022, and March 31st, 2023. Participant responses were recorded on tablets using REDCap and data quality review was undertaken weekly by the research coordinator (RC) and lead investigator [24].

### Financial toxicity outcome measures

#### Out of pocket costs

Each patient’s OOP costs were comprised of the following elements: consultation fees with specialists/generalists, procedural costs for surgical/medical interventions, radiology and imaging fees, admission costs based on length of stay and acuity of care (ICU vs. general wards), consumable products such as saline or blood, and death expenses. The per-item costs were obtained from KCMC administration and were up to date as of February 2023 (S1 Table). Total health expenditure was calculated using a conversion rate of 1 USD per 2,339 Tanzanian shillings (TZS).

Every patient saw at least one specialist–the emergency physician. Further specialist consults included surgery, cardiology, psychiatry, and urology. Generalists included dentists and primary care physicians. Physical/occupational therapy consultation was assessed separately. Procedurally, a subset of emergent care interventions was considered. Non-surgical procedures included intubation, nasopharyngeal airway placement, oxygen therapy, fluid administration, and blood transfusion. Surgical procedures ranged from surgical debridement with external fixation to tracheostomy; only procedural costs were included due to limited information. Imaging procedures included X-Rays, computerized tomography (CT) scans, magnetic resonance imaging (MRI), and ultrasound scans (USSs). For MRIs and USSs, specific type data was unavailable so the average cost for each imaging type was substituted. If it was unknown whether the imaging was unilateral or bilateral, the average cost of bilateral and unilateral scans was substituted. To calculate cost of stay, the length of stay at each care acuity level (Intensive Care Unit [ICU] vs. general ward) was used. The most basic level of accommodation on the shared wards was used to calculate costs as opposed to private room costs. For individuals who died during admission, we considered a death certificate, transportation from the wards to the morgue, and a body bag among the included expenses. Rates for Tanzanian citizens were used for all procedures as these were the least expensive and most appropriate for our sample population.

#### Catastrophic health expenditure

**Fig 2:**
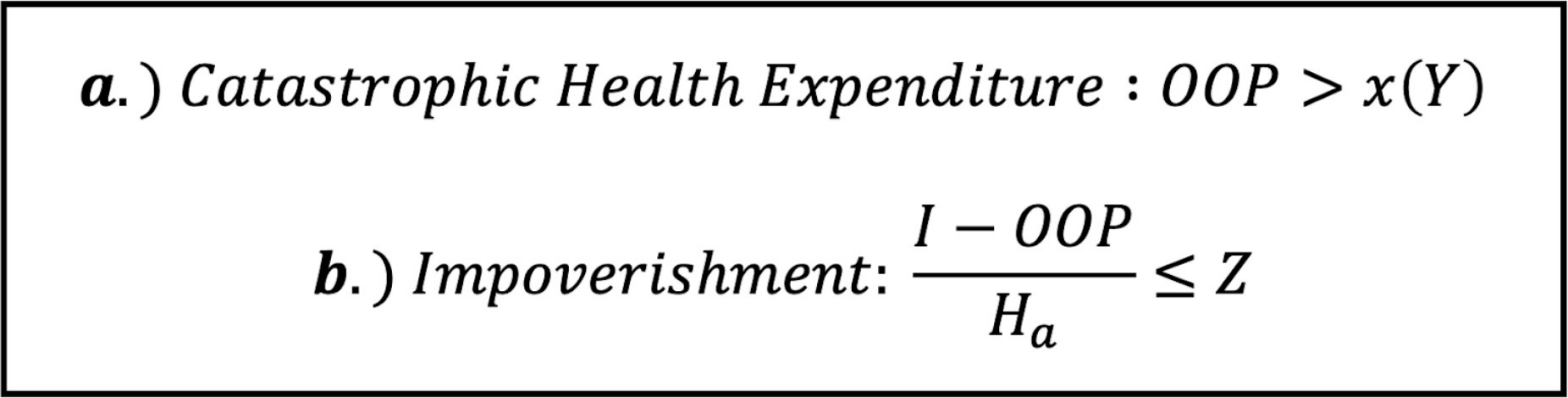
Equations used to calculate catastrophic health expenditure and impoverishment. a.) Catastrophic expenditure is defined as out-of-pocket costs exceeding 1) a fraction (x = 0.10 or 0.25) of a household’s annual total expenditure (Y = TE), or 2) a fraction (x = 0.40) of a household’s non-subsistence expenditure (Y = NSE). b.) Impoverishment was defined as a patient’s remaining per-capita income (after out-of-pocket costs) falling below one of the predefined poverty lines (Z = 49,320 TZS per month, $1.90 USD per person per day, or $3.20 USD per person per day).

Catastrophic expenditure (CHE) was calculated three different ways based on standards from the WHO (Fig 2) [25–27]. Total annual expenditure (TE) by household included monetary expenditures on all non-durable goods/services as well as the value use of durable goods and housing [28]. Non-subsistence expenditure (NSE) was calculated as TE minus subsistence expenditure [29]. Subsistence expenditure (a standard amount to cover basic needs) was quantified as the average amount spent on food by those households within our sample population who fell in the 45^th^-55^th^ percentiles of consumption [29]. If OOP costs were higher than any one threshold, the individual was considered as having experienced a catastrophic health expense.

#### Impoverishment

Impoverishment (IMP) also calculated three ways based on WHO guidelines as well as the Tanzanian national poverty line (Fig 2) [30–32]. Income (I) was defined on an annual, household basis. Household size was adjusted (H_a_) using the OECD-modified equivalence scale to find per adult-equivalent income [33]. If the value fell below a given poverty threshold, the individual was considered as having experienced an impoverishing expense.

### Data analysis

Participants were stratified by both CHE and IMP for descriptive analysis. Various factors were considered across the groups including sociodemographic, injury, and household characteristics. To further frame barriers and facilitators to care, the Three Delays Model was populated again stratifying by CHE and IMP. T-tests or ANOVAs were conducted for demographics between the presence/absence of the different types of financial toxicity yet could not be conducted for the varying levels of financial toxicity as those categories are not independent.

### Results

A total of 78 individuals who presented to KCMC’s EMD were included in the final sample. These individuals mostly consisted of males (83.5%) between the ages of 25–46 who experienced a road traffic injury (66.7%); this reflects existing literature for the Kilimanjaro region (Table 1) [34]. There were very few in-hospital deaths observed (2.6%) and most individuals were uninsured (83.3%). The median monthly adult-equivalent household income was 98,387 TZS or $42 USD while the median OOP cost was 476,793 TZS or $204 USD.

**Table 1:** Demographic, financial, and medical characteristics of the overall sample (n = 78).

***DEMOGRAPHIC INFORMATION***	
**Age** ^a^	32 [20.5]
**Sex**	
Female	16.7 (13)
Male	83.3 (65)
**Education**	
0–4 years	6.4 (5)
5–10 years	56.4 (44)
11+ years	34.6 (27)
**Education**	
Moshi Rural	61.5 (48)
Moshi Urban	3.8 (3)
Other	34.6 (27)
**Marital Status**	
Single	37.2 (29)
Married	47.4 (37)
Partner, Widowed, Separated	15.4 (12)
**Occupation**	
Farmer	35.9 (28)
Self-Employed	23.1 (18)
Skilled Employment	28.2 (22)
Other	12.8 (10)
**Number of dependents in household**	
0–2	47.4 (37)
3–5	37.2 (29)
6+	15.4 (12)
***FINANCIAL INFORMATION***	
**Insurance Status**	
Uninsured	83.3 (65)
National Health Insurance Fund	15.4 (12)
**Household income (monthly adult equivalent) (TZS)** ^a^	98,387.1 [136,842.1]
**Cost of hospital visit (TZS)** ^a^	476,792.7 [536,825]
***MEDICAL INFORMATION***	
**Mechanism of Injury**	
Blunt Force	10.3 (8)
Fall	16.7 (13)
Road Traffic Injury	66.7 (52)
Other	6.4 (5)
**Injury requires:**	
Hospitalization	80.8 (63)
Surgery	48.7 (38)

All values are listed as % (n) unless otherwise indicated.

^a^ Median [IQR]

In terms of CHE, 62.8% (n = 49) experienced some form of CHE (Table 2). Individuals who experienced a CHE were mostly farmers and tended to have larger household sizes and more dependents than those without a CHE. Individuals with a CHE also spent more days hospitalized; this was paired with a greater number who underwent at least 1 surgery (CHE: 63.2%; No CHE: 24.1%). Median monthly adult-equivalent income for individuals with any type of CHE was 2.52 times smaller than non-CHE households (62,500 TZS vs. 1157,895 TZS).

**Table 2:** Demographic, financial, and medical characteristics stratified by CHE status.

	Overall Comparison (n = 78)			CHE by Level		
	Any CHE (n = 49)	No CHE (n = 29)	p-value^b^	OOP ≥ 10% of TE (n = 46)	OOP ≥ 25% of TE (n = 22)	OOP ≥ 40% of NSE (n = 13)
***DEMOGRAPHIC INFORMATION***						
**Age** ^a^	33 [16]	30 [23]	0.924	33.5 [17.5]	38.5 [30.75]	32 [10]
**Sex**						
Female	14.3 (7)	20.7 (6)	0.675	15.2 (7)	22.7 (5)	14.3 (7)
Male	85.7 (42)	79.3 (23)		84.8 (39)	77.3 (17)	85.7 (42)
**Education**						
0–4 years	6.1 (3)	6.9 (2)	0.14	6.5 (3)	9.1 (2)	0 (0)
5–10 years	63.3 (31)	44.8 (13)		63 (29)	77.3 (17)	76.9 (10)
11+ years	30.6 (15)	41.4 (12)		30.4 (14)	13.6 (3)	23.1 (3)
**Region of Residence**						
Moshi Rural	57.1 (28)	69 (20)	0.224	56.5 (26)	63.6 (14)	69.2 (9)
Moshi Urban	2 (1)	6.9 (2)		2.2 (1)	0 (0)	0 (0)
Other	40.8 (20)	24.1 (7)		41.3 (19)	36.4 (8)	30.8 (4)
**Marital Status**						
Single	30.6 (15)	48.3 (14)	0.203	32.6 (15)	27.3 (6)	23.1 (3)
Married	53.1 (26)	37.9 (11)		54.3 (25)	54.5 (12)	53.8 (7)
Partner, Widowed, Separated	16.3 (8)	13.8 (4)		13 (6)	18.2 (4)	23.1 (3)
**Occupation**						
Farmer	40.8 (20)	27.6 (8)	0.819	41.3 (19)	59.1 (13)	46.2 (6)
Self-Employed	20.4 (10)	27.6 (8)		17.4 (8)	18.2 (4)	30.8 (4)
Skilled Employment	26.5 (13)	31 (9)		28.3 (13)	18.2 (4)	15.4 (2)
Other	12.2 (6)	13.8 (4)		13 (6)	4.5 (1)	7.7 (1)
**Number of dependents in household**						
0–2	46.9 (23)	48.3 (14)	0.61	43.5 (20)	45.5 (10)	46.2 (6)
3–5	34.7 (17)	41.4 (12)		37 (17)	31.8 (7)	46.2 (6)
6+	18.4 (9)	10.3 (3)		19.6 (9)	22.7 (5)	7.7 (1)
***FINANCIAL INFORMATION***						
**Insurance Status**						
Uninsured	87.8 (43)	75.9 (22)	0.241	87 (40)	90.9 (20)	100 (13)
National Health Insurance Fund	12.2 (6)	20.7 (6)		13 (6)	9.1 (2)	0 (0)
**Household income (monthly adult equivalent) (TZS)** ^a^	62,500 [126,000]	157,894.7 [219,015.3]	0.003**	61,250 [126,142.9]	23,905 [45,054.95]	62,500 [82,500]
**Cost of hospital visit (TZS)** ^a^	603,585.5 [323,935.5]	104,585.5 [205,000]	<0.001***	613,218 [254,306.2]	648,010 [253,912.5]	583,936 [438,564.5]
***MEDICAL INFORMATION***						
**Mechanism of Injury**						
**Blunt Force**	8.2 (4)	13.8 (4)	0.39	8.7 (4)	4.5 (1)	0 (0)
**Fall**	14.3 (7)	20.7 (6)		13 (6)	22.7 (5)	23.1 (3)
**Road Traffic Injury**	69.4 (34)	62.1 (18)		71.7 (33)	63.6 (14)	61.5 (8)
**Other**	8.2 (4)	3.4 (1)		6.5 (3)	9.1 (2)	15.4 (2)
**Injury requires:**						
**Hospitalization**	91.8 (45)	62.1 (18)	0.003**	95.7 (44)	95.5 (21)	84.6 (11)
**Surgery**	63.3 (31)	24.1 (7)	0.002**	67.4 (31)	63.6 (14)	53.8 (7)

All values are listed as % (n) unless otherwise indicated. Note: Responses of “unknown” are not displayed in this table but are included in percentage calculations.

^a^ Median [IQR]

^b^ p<0.05*; p<0.01**; p<0.001***

Out of the whole sample, 85.9% (n = 67) experienced some form of IMP because of their hospital OOP costs (Table 3). Individuals experiencing an impoverishing expense had fewer years of education (68.7% with ≥10 years) and more of those individuals underwent at least 1 surgery (IMP: 50.8%; No IMP: 33.3%). Individuals experiencing an impoverishing expense also tended to have larger household sizes and more dependents than those without impoverishment–none of the non-impoverished households had ≥6 dependents. Median monthly adult-equivalent income of impoverished households was 4.26 times smaller than in non-impoverished households (93,750 TZS vs. 400,000 TZS).

**Table 3:** Demographic, financial, and medical characteristics stratified by IMP status.

	Overall Comparison (n = 78)			IMP by Level		
	Any IMP (n = 67)	No IMP (n = 9)	p-value^b^	National poverty line (n = 28)	≤ $1.90 USD pp/pd (n = 61)	≤ $3.20 USD pp/pd (n = 67)
***DEMOGRAPHIC INFORMATION***						
**Age** ^a^	34 [21]	26 [4]	0.1	39 [27]	35 [20]	34 [21]
**Sex**						
Female	16.4 (11)	22.2 (2)	0.74	23.5 (4)	18 (11)	16.4 (11)
Male	83.6 (56)	77.8 (7)		76.5 (13)	82 (50)	83.6 (56)
**Education**						
0–4 years	7.5 (5)	0 (0)	0.185	11.8 (2)	8.2 (5)	7.5 (5)
5–10 years	61.2 (41)	33.3 (3)		82.4 (14)	65.6 (40)	61.2 (41)
11+ years	29.9 (20)	55.6 (5)		5.9 (1)	24.6 (15)	29.9 (20)
**Region of Residence**						
Moshi Rural	58.2 (39)	88.9 (8)	0.178	52.9 (9)	57.4 (35)	58.2 (39)
Moshi Urban	3 (2)	11.1 (1)		0 (0)	1.6 (1)	3 (2)
Other	38.8 (26)	0 (0)		47.1 (8)	41 (25)	38.8 (26)
**Marital Status**						
Single	28.4 (19)	88.9 (8)	0.043*	29.4 (5)	26.2 (16)	28.4 (19)
Married	53.7 (36)	11.1 (1)		58.8 (10)	54.1 (33)	53.7 (36)
Partner, Widowed, Separated	17.9 (12)	0 (0)		11.8 (2)	21.3 (13)	17.9 (12)
**Occupation**						
Farmer	40.3 (27)	11.1 (1)	0.015*	76.5 (13)	41 (25)	40.3 (27)
Self-Employed	23.9 (16)	22.2 (2)		5.9 (1)	24.6 (15)	23.9 (16)
Skilled Employment	26.9 (18)	33.3 (3)		17.6 (3)	27.9 (17)	26.9 (18)
Other	9 (6)	33.3 (3)		0 (0)	6.6 (4)	9 (6)
**Number of dependents in household**						
0–2	43.3 (29)	66.7 (6)	0.297	41.2 (7)	42.6 (26)	43.3 (29)
3–5	38.8 (26)	33.3 (3)		29.4 (5)	37.7 (23)	38.8 (26)
6+	17.9 (12)	0 (0)		29.4 (5)	19.7 (12)	17.9 (12)
***FINANCIAL INFORMATION***						
**Insurance Status**						
Uninsured	85.1 (57)	66.7 (6)	0.572	94.1 (16)	88.5 (54)	85.1 (57)
National Health Insurance Fund	13.4 (9)	33.3 (3)		5.9 (1)	9.8 (6)	13.4 (9)
**Household income (monthly adult equivalent) (TZS)** ^a^	93,750 [106,639.9]	400,000 [263,157.9]	<0.001***	15,789 [19,230.77]	83,333 [105,357.1]	93,750 [106,639.9]
**Cost of hospital visit (TZS)** ^a^	529,935.5 [527,250]	230,000 [240,935.5]	0.253	549,936 [297,064.5]	520,727 [540,850]	529,935.5 [527,250]
***MEDICAL INFORMATION***						
**Mechanism of Injury**						
**Blunt Force**	10.4 (7)	11.1 (1)	0.482	5.9 (1)	11.5 (7)	10.4 (7)
**Fall**	16.4 (11)	22.2 (2)		29.4 (5)	18 (11)	16.4 (11)
**Road Traffic Injury**	67.2 (45)	55.6 (5)		47.1 (8)	63.9 (39)	67.2 (45)
**Other**	4.5 (3)	11.1 (1)		17.6 (3)	6.6 (4)	4.5 (3)
**Injury requires:**						
**Hospitalization**	82.1 (55)	66.7 (6)	0.427	82.4 (14)	82 (50)	82.1 (55)
**Surgery**	50.7 (34)	33.3 (3)	0.617	35.3 (6)	49.2 (30)	50.7 (34)

All values are listed as % (n) unless otherwise indicated. Responses of “unknown” are not displayed in this table but are included in percentage calculations. Two individuals were excluded from all impoverishment columns in this table as they declined to provide income information. Note: pp/pd = per person, per day

^a^ Median [IQR]

^b^ p<0.05*; p<0.01**; p<0.001***

**Fig 3:**
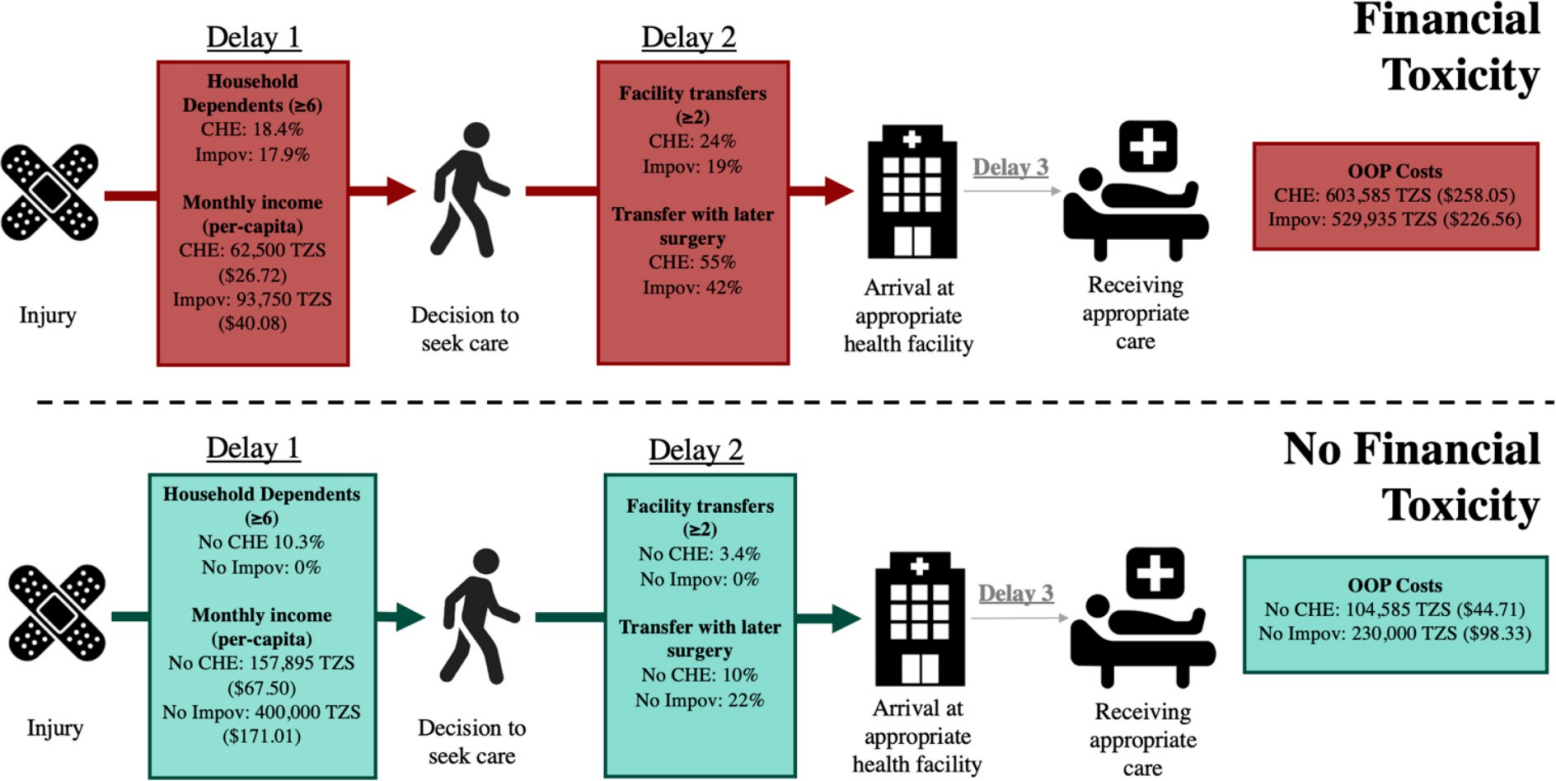
The Three Delays Model populated for injury patients who seek emergency care at KCMC stratified by the experience of financial toxicity.

In terms of delays to care, individuals who experienced CHE or IMP had a greater number of facility transfers, particularly those with later surgery (Fig 3). Factors classified under Delay 1 (the decision to seek care) and Delay 2 (reaching the appropriate care facility) seemed to be hallmarks of the prehospital care pathway for individuals who experienced some form of financial toxicity (Fig 3). The Third Delay appeared less impactful as most individuals across CHE and IMP categories reported that it took “minutes” to receive care once arriving at KCMC (CHE: 90%; No CHE: 93%; IMP: 94%; No IMP: 89%). OOP costs were notably higher in those groups who experienced financial toxicity—for individuals experiencing CHE or IMP, median OOP costs were 5.77 times and 2.30 times larger, respectively, than those who did not experience financial toxicity (Fig 3).

## Discussion

To push towards UHC, reductions of health inequities, and improved health outcomes for all, the WHO considers both CHE and IMP to be critical indicators for achieving the associated Sustainable Development Goals [35]. In this study, injury registry data and patient reports indicated that individuals experiencing financial toxicity had larger households, more dependents, and lower baseline incomes. These differences paint the picture of households which are financially constrained before becoming injured, with more mouths to feed and individuals who need additional care. Another study in the Kilimanjaro region demonstrated that even the thought of OOP expenses associated with a hospital visit led to patients delaying care, with specific exacerbations in Delay 1 [22]. Increases in Delay 1 have also been associated with other factors such as loss of income and childcare—critical points of consideration when delaying care [5, 7].

Once deciding to seek care, individuals who experience financial toxicity reported more facility transfers and more frequent transfers with eventual surgery. As shown by a study in Kenya, injury patients who underwent hospital transfers had a higher odds of in-hospital mortality in comparison to those who were able to directly access care at their ultimate hospital destination [36]. This difference could be because the patients who need transfer are sicker from the beginning, yet it could also be due to the delays in their definitive care—it remains unclear which factor is more influential. In addition, the semi-rigid healthcare system in Tanzania begets transfers for those who are dependent on the NHIF and must first seek care at a lower-level facility for a referral to avoid additional costs, even if the receiving facility does not have the appropriate resources [37]. Therefore, this higher surgical burden interacting with a higher facility transfer rate could suggest systematic delays that impact health system pathways. This mirrors findings in high-income countries (HIC), yet prehospital trauma systems in HICs reduce morbidity and mortality of severely injured patients by transporting immediately to higher-level hospitals without engaging in an interfacility transfer/referral system [37]. The absence of any prehospital trauma system in the Kilimanjaro region suggests exacerbations in Delay 2 as shown by a higher surgical burden and facility transfer rate.

In our study, we did not observe a substantial difference in Delay 3 between those experiencing financial toxicity and those who do not. This could be due to confounding effects like the quality of care at KCMC or an increased availability of resources/personnel as KCMC is the tertiary care facility for the region. However, our study is unique in presenting and assessing all three delays of the Three Delays model rather than focusing on hospital characteristics (Delay 3). A systematic review of the Three Delays Model applied to trauma systems in LMICs found that the delay in receiving appropriate care (Delay 3) was the most frequently assessed in the literature [18]. However, our study shows there could be significant barriers to emergency care due to individual factors which can influence decision-making and individual paths to care.

The main limitations of this study are subject selection bias, inability to consider full impact of NHIF, and the use of incomplete financial measures for OOP costs. The use of an injury registry for sampling likely introduces some subject selection bias via underrepresentation of the most severe injury cases who did not make it to KCMC or died during transport, as well as an underrepresentation of the most financially constrained individuals. Those individuals with severe injuries are less likely to have made it to an outside hospital, been stable enough for transfer, and successfully been transferred to KCMC to be included in the injury registry. Similarly, those who have the fewest resources to begin with are unlikely to be able to pay to reach KCMC and are less likely to appear in the trauma registry. Secondly, as the NHIF has varying coverage levels depending on a patient’s job classification, we calculated OOP expenses as they would be charged pre-reimbursement from the NHIF as the trauma registry did not contain patient job classification data [38]. However, this only applied to a small portion of the study population as the vast majority were uninsured. Finally, as the costs were calculated based on itemized lists of procedures and expenses from a limited definition of healthcare expense, it is likely that the final calculated healthcare expenditures were underestimated due to missed expenses. Therefore, our results represent conservative estimates for financial toxicity within this patient population.

To support healthcare utilization and protect households from financial toxicity originating from high OOP costs, it is critical to expand health coverage for emergency care [39]. However, as we found in our study, the high OOP costs could originate due to inequities along the pre-hospital pathway; expansion of UHC should extend to financial protection from in-hospital OOP costs in addition to measures that reduce pre-hospital delays. Especially in a Tanzanian system wherein the current National Health Insurance Fund (NHIF) lapses when an individual is unemployed or has more than four legal dependents, it is essential to expand protections for those at-risk groups where baseline financial burden is high [40]. Our findings could suggest that differences in OOP costs originate before an individual even decides to seek care for their emergency, exacerbating underlying inequities and disproportionately increasing the burden for the poorest of the poor. We suggest several policy recommendations to protect individuals who experience acute injury from financial toxicity:

- Scale-up and expand UHC mechanisms to include additional protection for acute injuries and other emergent conditions wherein reducing delays to care is critical for health outcomes.
- Systematically focus interventions on households who the NHIF does not cover completely which could be at higher risk for financial toxicity such as those which are rural, have breadwinners who are unemployed, or with high numbers of dependents.
- Include provisions for potential pre-hospital delays and confounders of the pre-hospital care pathway, such as emergent transportation and specialized surgical interventions.

## Supporting information

S1 Table Complete survey administered to participants of this study. (DOCX)

S2 Table All expense types incurred by patients at KCMC. (DOCX)
