# Supplementary material for: Financial toxicity and acute injury in the Kilimanjaro region: An application of the Three Delays Model
Source: PLoS One. 2024 Aug 30;19(8):e0308539. doi: 10.1371/journal.pone.0308539 (PMC11364231; doi:10.1371/journal.pone.0308539)
Supplement: S1 Table — (DOCX) [file pone.0308539.s001.docx]

**S1 Table. Complete survey administered to participants of this study.**

| **Subsection** | **Question (Swahili)** | **Answer Choices (if multiple choice)** | **Question (English)** |
| --- | --- | --- | --- |
| Demographics | Nambari ya Rekodi ya REDCap: | - | REDCap Record Number: |
|  | Kitambulisho cha Usajili wa Trauma: | - | Trauma Registry ID: |
|  | Mgonjwa ana umri gani? | - | How old is the patient? |
|  | Jinsia ya mgonjwa ni nini? | 0, Kiume (Male) \| 1, Kike (Female) | What is the patient's sex? |
|  | Mgonjwa alifika KCMC tarehe ngapi (DD-MM-YYYY)? | - | On what date did the patient arrive at KCMC (DD-MM-YYYY)? |
|  | Je, mshiriki huyu ni mgonjwa au mwanafamilia? | 0, Mgonjwa (Patient) \| 1, Mwanafamilia (Family Member) | Is this participant a patient or a family member? |
| Health Utilization Barriers | Kama ndio, wewe/mwanafamilia wako amekuwa na dalili kwa muda gani kabla ya kutafuta huduma ya afya? | 1, Siku (Days) \| 2, Masaa (Hours) \| 3, Dakika (Minutes) \| 999, Haijulikani/Sikumbuki (Unknown/Can't Remember) | How long did you/your family member have symptoms before seeking care? |
|  | Siku: | - | Days: |
|  | Masaa: | - | Hours: |
|  | Dakika: | - | Minutes: |
|  | Ulienda wewe/mwanafamilia mara ya kwanza? | 1, Zahanati (Dispensary) \| 2, Kituo cha afya (Health center) \| 3, Kliniki (Clinic) \| 4, Hospitali (Hospital) \| 5, Jina Hospitali (Name of Hospital) \| 6, Mponyaji wa kiimani (Faith healer) \| 7, Mganga wa kienyeji (Traditional healer) \| 8, Duka la dawa (Pharmacy) \| 9, Duka la kawaida (Small shop over-the-counter medications) \| 999, Nyingine (Other) | Where did you/your family member go initially? |
|  | Nyingine: | - | Other: |
|  | Kwa wewe/mwanafamilia ulichagua kwenda hapo? | 1, Ni kituo cha karibu (Closest facility) \| 2, Mazoea (Familiarity) \| 3, Gharama (Cost) \| 4, Ubora wa huduma (Quality of care) \| 5, Huduma kutoka kwa daktari fulani (Care of a specific doctor) \| 6, Kuna bima (Covered by insurance) \| 7, Upatikanaji wa utaalamu (Available specialty) \| 999, Haijulikani/Sikumbuki (Unknown/Can't Remember) | Why did you/your family member choose to go there? |
|  | Wewe/Mwanafamilia ulifikaje hapo? | 1, Gari (binafsi) (Car private) \| 2, Gari (taxi) (Car taxi) \| 3, Bajaji (Bajaji) \| 4, Pikipiki (Motorcycle) \| 5, Daladala (dala dala) \| 6, Baiskeli (Bicycle) \| 7, Basi kubwa (Large bus) \| 8, Kutembea (Walking) \| 9, Gari (la wagonjwa) (Ambulance) \| 10, Haijulikani/Sikumbuki (Unknown/Can't remember) \| 11, Gari (la polisi) (Police car) | How did you/your family member get there? |
|  | Ilikuchukua muda gani wewe/mwanafamilia kufika hapo? | 1, Siku (Days) \| 2, Masaa (Hours) \| 3, Dakika (Minutes) | How long did it take you/your family member to get there? |
|  | Siku: | - | Days: |
|  | Masaa: | - | Hours: |
|  | Dakika: | - | Minutes: |
|  | Je, wewe/mwanafamilia ulihamishiwa katika kituo kingine? | 1, Ndio (Yes) \| 2, Hapana (No) | Were you/your family member transferred to another facility? |
|  | Kama ndiyo, wapi? | - | If yes, where? |
|  | Je, wewe/mwanafamilia umehamishwa mara ngapi kabla hujafika kituo cha mwisho? (Ikiwa haijulikani/hawezi kukumbuka, chapa 999) | - | How many times were you/your family member transferred before your/their final destination? (If, unknown/can't remember, type 999) |
|  | Wewe/Mwanafamilia ulifikaje hapo? | 1, Gari (binafsi) (Car private) \| 2, Gari (taxi) (Car taxi) \| 3, Bajaji (Bajaji) \| 4, Pikipiki (Motorcycle) \| 5, Daladala (dala dala) \| 6, Baiskeli (Bicycle) \| 7, Basi kubwa (Large bus) \| 8, Kutembea (Walking) \| 9, Gari (la wagonjwa) (Ambulance) \| 10, Haijulikani/Sikumbuki (Unknown/Can't remember) \| 11, Gari (la polisi) (Police car) | How did you/your family member get there? |
|  | Ilikuchukua muda gani wewe/mwanafamilia tangu kuamua kutafuta huduma mpaka kufika katika kituo cha mwisho? | 1, Siku (Days) \| 2, Masaa (Hours) \| 3, Dakika (Minutes) | How long from when you/your family member decided to seek care to when you/they reached the final facility? |
|  | Siku: | - | Days: |
|  | Masaa: | - | Hours: |
|  | Dakika: | - | Minutes: |
|  | Ilikuchukua muda gani wewe/mwanafamilia tangu kufika mpaka umuone daktarin au nesi katika kituo cha mwisho? | 1, Siku (Days) \| 2, Masaa (Hours) \| 3, Dakika (Minutes) | How long from the time you/your family member arrived until you/they were seen by a doctor or nurse at the final facility? |
|  | Siku: | - | Days: |
|  | Masaa: | - | Hours: |
|  | Dakika: | - | Minutes: |
| Emergency Scenarios | Kisa cha 1: Mama yako ambaye ni mzee anaamka na hawezi kusogeza mkono au mguu wake wa kulia. Uso wake umeshuka upande mmoja na anachanganya maneno. Unafikiri hii ni mbaya kiasi gani? | 1, Sio mbaya kabisa (Not at all severe) \| 2, Inawezekana ni mbaya (Possibly severe) \| 3, Ni mbaya kiasi (Somewhat severe) \| 4, Ni mbaya (Severe) \| 5, Ni mbaya sana (Very severe) | Scenario 1: Your older mother wakes up and can not move her right arm or leg. Her face is drooping and her speech is slurred. How severe do you think this is? |
|  | Je, ungetafuta huduma kwa ajili ya hii? | 1, Ndio (Yes) \| 2, Hapana (No) | Would you seek care for this? |
|  | Kama ni ndiyo, ungetafuta wapi huduma? | 1, Zahanati (Dispensary) \| 2, Kituo cha afya (Health center) \| 3, Kliniki (Clinic) \| 4, Hospitali (Hospital) \| 5, Jina Hospitali (Name of Hospital) \| 6, Mponyaji wa kiimani (Faith healer) \| 7, Mganga wa kienyeji (Traditional healer) \| 8, Duka la dawa (Pharmacy) \| 9, Duka la kawaida (Small shop over-the-counter medications) \| 999, Nyingine (Other) | If yes, where would you seek care? |
|  | Nyingine: | - | Other: |
|  | Ungetafuta huduma kwa haraka kiasi gani? | 1, Mara moja/bila kukawia (Immediately) \| 2, Chini ya siku moja (Less than a day) \| 3, Kati ya siku 1-2 (Between 1-2 days) \| 4, siku 3 au zaidi (3 days or more) | How quickly would you seek care? |
|  | Kisa cha 2: Mtoto wako wa kiume mwenye miaka 14 ana maumivu makali ya tumbo na anatapika na kulia kwa muda mrefu bila kuacha. Hajala chakula kwa siku 3 na amekataa maji kwa siku. Unafikiri hii ni mbaya kiasi gani? | 1, Sio mbaya kabisa (Not at all severe) \| 2, Inawezekana ni mbaya (Possibly severe) \| 3, Ni mbaya kiasi (Somewhat severe) \| 4, Ni mbaya (Severe) \| 5, Ni mbaya sana (Very severe) | Scenario 2: Your 14-year-old son has very bad pain in his stomach and can not stop vomiting and crying. He has not eaten food in 3 days and refuses water for 2 days. How severe do you think this is? |
|  | Je, ungetafuta huduma kwa ajili ya hii? | 1, Ndio (Yes) \| 2, Hapana (No) | Would you seek care for this? |
|  | Kama ni ndiyo, ungetafuta wapi huduma? | 1, Zahanati (Dispensary) \| 2, Kituo cha afya (Health center) \| 3, Kliniki (Clinic) \| 4, Hospitali (Hospital) \| 5, Jina Hospitali (Name of Hospital) \| 6, Mponyaji wa kiimani (Faith healer) \| 7, Mganga wa kienyeji (Traditional healer) \| 8, Duka la dawa (Pharmacy) \| 9, Duka la kawaida (Small shop over-the-counter medications) \| 999, Nyingine (Other) | If yes, Where would you seek care? |
|  | Nyingine: | - | Other: |
|  | Ungetafuta huduma kwa haraka kiasi gani? | 1, Mara moja/bila kukawia (Immediately) \| 2, Chini ya siku moja (Less than a day) \| 3, Kati ya siku 1-2 (Between 1-2 days) \| 4, siku 3 au zaidi (3 days or more) | How quickly would you seek care? |
|  | Kisa cha 3: Mtoto wako wa kike wa miezi 4 anaharisha, kutapika na ana homa kali kwa siku 2. Analala siku nzima, hanywi maziwa na sasa hivi huwezi kumuamsha. Unafikiri hii ni mbaya kiasi gani? | 1, Sio mbaya kabisa (Not at all severe) \| 2, Inawezekana ni mbaya (Possibly severe) \| 3, Ni mbaya kiasi (Somewhat severe) \| 4, Ni mbaya (Severe) \| 5, Ni mbaya sana (Very severe) | Scenario 3: Your 4-month-old has diarrhea, vomiting and very high fever for 2 days. She is sleeping all day, not drinking milk, and now you can not wake her up. How severe do you think this is? |
|  | Je, ungetafuta huduma kwa ajili ya hii? | 1, Ndio (Yes) \| 2, Hapana (No) | Would you seek care for this? |
|  | Kama ni ndiyo, ungetafuta wapi huduma? | 1, Zahanati (Dispensary) \| 2, Kituo cha afya (Health center) \| 3, Kliniki (Clinic) \| 4, Hospitali (Hospital) \| 5, Jina Hospitali (Name of Hospital) \| 6, Mponyaji wa kiimani (Faith healer) \| 7, Mganga wa kienyeji (Traditional healer) \| 8, Duka la dawa (Pharmacy) \| 9, Duka la kawaida (Small shop over-the-counter medications) \| 999, Nyingine (Other) | If yes, Where would you seek care? |
|  | Nyingine: | - | Other: |
|  | Ungetafuta huduma kwa haraka kiasi gani? | 1, Mara moja/bila kukawia (Immediately) \| 2, Chini ya siku moja (Less than a day) \| 3, Kati ya siku 1-2 (Between 1-2 days) \| 4, siku 3 au zaidi (3 days or more) | How quickly would you seek care? |
|  | Kisa cha 4: Baba yako anaamka na maumivu makali sana ya kifua na anahema kwa shida. Unafikiri hii ni mbaya kiasi gani? | 1, Sio mbaya kabisa (Not at all severe) \| 2, Inawezekana ni mbaya (Possibly severe) \| 3, Ni mbaya kiasi (Somewhat severe) \| 4, Ni mbaya (Severe) \| 5, Ni mbaya sana (Very severe) | Scenario 4: Your father wakes up with very bad chest pain and trouble breathing. How severe do you think this is? |
|  | Je, ungetafuta huduma kwa ajili ya hii? | 1, Ndio (Yes) \| 2, Hapana (No) | Would you seek care for this? |
|  | Kama ni ndiyo, ungetafuta wapi huduma? | 1, Zahanati (Dispensary) \| 2, Kituo cha afya (Health center) \| 3, Kliniki (Clinic) \| 4, Hospitali (Hospital) \| 5, Jina Hospitali (Name of Hospital) \| 6, Mponyaji wa kiimani (Faith healer) \| 7, Mganga wa kienyeji (Traditional healer) \| 8, Duka la dawa (Pharmacy) \| 9, Duka la kawaida (Small shop over-the-counter medications) \| 999, Nyingine (Other) | If yes, Where would you seek care? |
|  | Nyingine: | - | Other: |
|  | Ungetafuta huduma kwa haraka kiasi gani? | 1, Mara moja/bila kukawia (Immediately) \| 2, Chini ya siku moja (Less than a day) \| 3, Kati ya siku 1-2 (Between 1-2 days) \| 4, siku 3 au zaidi (3 days or more) | How quickly would you seek care? |
|  | Kisa cha 5: Uko kwenye ajali mbaya ya pikipiki na umegonga kichwa chako. Mfupa wa paja umevunjika na hauwezi kuhema vizuri. Unafikiri hii ni mbaya kiasi gani? | 1, Sio mbaya kabisa (Not at all severe) \| 2, Inawezekana ni mbaya (Possibly severe) \| 3, Ni mbaya kiasi (Somewhat severe) \| 4, Ni mbaya (Severe) \| 5, Ni mbaya sana (Very severe) | Scenario 5: You are in a bad motorcycle crash and hit your head. Your left arm is broken and you can not breathe well. How severe do you think this is? |
|  | Je, ungetafuta huduma kwa ajili ya hii? | 1, Ndio (Yes) \| 2, Hapana (No) | Would you seek care for this? |
|  | Kama ni ndiyo, ungetafuta wapi huduma? | 1, Zahanati (Dispensary) \| 2, Kituo cha afya (Health center) \| 3, Kliniki (Clinic) \| 4, Hospitali (Hospital) \| 5, Jina Hospitali (Name of Hospital) \| 6, Mponyaji wa kiimani (Faith healer) \| 7, Mganga wa kienyeji (Traditional healer) \| 8, Duka la dawa (Pharmacy) \| 9, Duka la kawaida (Small shop over-the-counter medications) \| 999, Nyingine (Other) | If yes, Where would you seek care? |
|  | Nyingine: | - | Other: |
|  | Ungetafuta huduma kwa haraka kiasi gani? | 1, Mara moja/bila kukawia (Immediately) \| 2, Chini ya siku moja (Less than a day) \| 3, Kati ya siku 1-2 (Between 1-2 days) \| 4, siku 3 au zaidi (3 days or more) | How quickly would you seek care? |
| Health Needs | Je, kama wewe au mwanafamilia wako dharuranyingine ya afya, je ni kitu gani cha kwanza ambacho kitafanya kufika hospitali iwe rahisi? | 1, Kupata usafiri (Provision of transportation) \| 2, Kupunguza gharama za huduma ya afya (Lower costs of healthcare) \| 3, Kuwa na familia au rafiki wa kukusaidia (Family or friend available to help) \| 4, Uelewa mzuri zaidi wa uwezo wa hospitali (Better understanding of hospital capabilities ) \| 5, Kuwa na kituo cha afya au hospitali karibu zaidi na nyumbani (Closer facility in my community) \| 6, Barabara nzuri (Better roads) \| 7, Uboreshaji wa masaa katika vituo vya karibu (Improved hours of local facility) | If you or a family member has another health emergency, what is the number one thing that would make it easier to go to the hospital? |
|  | Una uweo wa kutumia simu yako wakati wote? | 1, Ndio (Yes) \| 2, Hapana (No) | Do you have a cell phone or access to a cell phone for most of the day? |
|  | Kama hapana, kwa nini? | 1, Hakuna mtandao kwetu (Poor coverage) \| 2, Chaji ya betri (Battery charge) \| 3, Kukosa vocha/dakika za mongezi (Lack of air/talk time) \| 4, Iliyopotezwa (Lost it) \| 5, Usimiliki (Don't own one) | If no, why not? |
|  | Je, unasaidia wategemezi wangapi katika kaya yako (watu ambao hawafanyi kazi na/au wanakutegemea ili uendelee kuishi kama vile watoto, wazee, n.k.)? | - | How many dependents do you support in your household (individuals who do not work and/or depend on your for survival such as children, elders, etc.)? |
| Monthly Income | Je, mshiriki alikataa kutoa majibu kwa fomu hii? | 1, Ndio (Yes) \| 2, Hapana (No) | Did the participant refuse to provide answers for this form? |
|  | Kaya yako ina ukubwa gani, ikijumuishwa na wewe mwenyewe (ni watu wangapi kwa kawaida wanaishi katika nyumba yako)? | - | What is the size of your household, including yourself (how many members normally live in your house)? |
|  | Mtu anayetengeneza pesa nyingi zaidi kwenye kaya yako anafanya kazi gani? (mtunzaji mkuu wa familia)? | 1, Mwanafunzi (Student) \| 2, Haja ajiriwa (Unemployed) \| 3, Mtaalamu (Professional) \| 4, Ajira yenye ujuzi (Skilled employment) \| 5, Amejiajiri (Self-employed) \| 6, Mkulima (Farmer) \| 7, Nyingine (Other) | What occupation is held by the person who makes the most money in your household (primary breadwinner?)? |
|  | Nyingine: | - | Other: |
|  | Kipato cha kaya yako ni kiasi gani kwenye mwezi wa kawaida (TZS)? | - | How much does your household earn in an average month? |
| Monthly Expenditure | Je, mshiriki alikataa kutoa majibu kwa fomu hii? | 1, Ndio (Yes) \| 2, Hapana (No) | Did the participant refuse to provide answers for this form? |
|  | Je, kaya yako inatumia pesa kiasi gani kwenye chakula, maji, juisi, au soda za kunywa kwa mwezi wa kawaida (TZS)? | - | How much money does your household spend on food to eat, water, juice, or soda to drink in an average month? |
|  | Je, kaya yako inatumia pesa kiasi gani kwenye mifugo (ikiwa ni pamoja na ng'ombe, nguruwe, mbuzi, kondoo, kuku etc.) kwa mwezi wa kawaida (TZS)? | - | How much money does your household spend on livestock (including cows, pigs, goats, sheep, chickens etc.) in an average month? |
|  | Ukitoa gharama za wakati huu ukiwa hospitalini, kwa kawaida kaya yako inatumia pesa kiasi gani kwenye huduma ya afya, ikiwa ni pamoja na dawa, gharama za kumuona daktari au kuhudhuria hospitali, gharama za mganga wa kienyeji kwa mwezi wa kawaida (TZS)? | - | Excluding this hospital course, how much money does your household usually spend on health care, including medicines, fees for doctors or hospital visits, fees for traditional healers in an average month? |
|  | Kaya yako inatumia pesa kiasi gani kwenye usafiri kwa mwezi wa kawaida? | - | How much money does your household spend on transportation in an average month? |
|  | Je, kaya yako inatumia pesa kiasi gani kwenye gharama za shughuli za kijamii kama harusi, sherehe, matamasha au mazishi kwa mwezi wa kawaida (TZS)? | - | How much money does your household spend on expenses for public gatherings such as weddings, parties, festivals or burials in an average month? |
|  | Je, kaya yako inatumia pesa kiasi gani kwenye vitu vingine vya nyumbani kama nguo, maboresho ya nyumba n.k. kwa mwezi wa kawaida (TZS)? | - | How much money does your household spend on other household matters such as clothes, improvements to your house etc. in an average month? |
|  | Je, kaya yako inatumia pesa kiasi gani kwenye elimu kwa mwaka kwa watoto wote unaowalipia, ikiwa ni pamoja na ada, vitabu, sare, usafiri na ada ya bweni (TZS)? | - | How much money does your household spend on education per year for all the children you support, including school fees, books, uniforms, transport and boarding fees? |
|  | Je, unategemea kutumia pesa kiasi gani kwenye hudhurio hili la Idara ya dharura/hospitali (TZS)? | - | How much money do you expect to spend on this visit to the emergency department/hospital? |
|  | Je, mpango wako ni upi kwenye kulipia hudhurio hili la idara ya dharura/hospitali (TZS)? | 1, Malipo binafsi ya pesa taslimu/akiba (Cash personal payment/savings ) \| 2, Pesa kutoka kwa ndugu (Money from relatives) \| 3, Bima (Insurance) \| 4, Msaada wa hospitali/msamaha (Hospital support/exemption) \| 5, Nyingine (Other) | How do you plan on paying for this visit to the emergency department/hospital? |
|  | Nyingine: | - | Other: |
|  | Je una matumizi mengine kwa mwezi? | 1, Ndio (Yes) \| 2, Hapana (No) | Do you have other monthly expenditures? |
|  | Kama ni hivyo, ni yapi? | 1, Huduma (umeme, maji, n.k.) (Utilities (electricity, water, etc.)) \| 2, Simu / Mtandao (Phone/Internet) \| 3, Utunzaji wa kipenzi kama mbwa au paka (Pet care) \| 4, Ulipaji wa deni (Debt repayment) \| 5, Nyingine (Other) | If so, what are they? |
|  | Je, kaya yako hutumia pesa ngapi kwa huduma kwa mwezi wa kawaida (TZS)? | - | How much money does your household spend on utilities in an average month? |
|  | Je, kaya yako inatumia pesa ngapi kwenye simu/mtandao kwa mwezi wa kawaida (TZS)? | - | How much money does your household spend on phone/internet in an average month? |
|  | Je, kaya yako hutumia pesa ngapi kwa utunzaji wa wanyama kama mbwa au paka kwa mwezi wa kawaida (TZS)? | - | How much money does your household spend on pet care in an average month for animals like dogs and cats? |
|  | Je, kaya yako hutumia pesa ngapi kulipa deni kwa mwezi wa kawaida (TZS)? | - | How much money does your household spend on debt repayment in an average month? |
|  | Je, ni gharama gani zingine za kila mwezi ambazo hazijatajwa vinginevyo (TZS)? | - | What are your other monthly expenses not otherwise named? |
|  | Je, kaya yako hutumia pesa ngapi kwa matumizi mengine kwa mwezi wa kawaida (TZS)? | - | How much money does your household spend on these other expenses in an average month? |
