# Supplementary material for: Financial toxicity and acute injury in the Kilimanjaro region: An application of the Three Delays Model
Source: PLoS One. 2024 Aug 30;19(8):e0308539. doi: 10.1371/journal.pone.0308539 (PMC11364231; doi:10.1371/journal.pone.0308539)
Supplement: S2 Table — (DOCX) [file pone.0308539.s002.docx]

# S2 Table. All expense types incurred by patients at KCMC.

| **Procedure Name** | **Price (TZS)** |
| --- | --- |
| **Consultation** | |
| Generalist | 10,000 |
| PT/OT | 8,000 |
| Specialist | 25,000 |
| **Surgical Procedures** | |
| Acetabular ORIF | 250,000 |
| Distal Tibia (Pilon) LT + LII | 350,000 |
| Femoral Shaft (Diaphyseal)* | 333,333·30 |
| ORIF – otherwise specified | 350,000 |
| Laparotomy | 350,000 |
| ORIF* | 342,727·30 |
| Surgical Debridement with External Fixator | 350,000 |
| Surgical Debridement with Implant | 300,000 |
| Surgical Debridement without External Fixator | 350,000 |
| Tibia Transtibial Amputation | 350,000 |
| Tracheostomy + tracheostomy tube | 600,000 |
| Acetabular Reconstruction | 1,100,000 |
| **Other Procedures** | |
| Intubation + endotracheal tube | 105,200 |
| NPA + airway guedel | 3,300 |
| Oxygen Therapy* | 23,650 |
| Fluid | 2,000 |
| Blood transfusion + Blood grouping/Rh typing & cross matching + Comb’s test | 12,000 |
| **X-Rays** | |
| Ankle (unilateral) | 20,000 |
| Ankle (unknown sidedness) | 30,000 |
| Chest | 20,000 |
| Clavicle (unknown sidedness) | 30,000 |
| Femur (unilateral) |  |
| Femur (unknown sidedness) | 30,000 |
| Foot (unilateral) | 20,000 |
| Foot (unknown sidedness) | 30,000 |
| Forearm | 20,000 |
| Hand | 20,000 |
| Knee | 20,000 |
| Pelvis | 20,000 |
| Radius/Ulna (unilateral) | 20,000 |
| Radius/Ulna (unknown sidedness) | 30,000 |
| Shoulder (unknown sidedness) | 30,000 |
| Skull | 20,000 |
| Spine (unknown region) | 20,000 |
| Thoracic/Lumbar Spine | 40,000 |
| Tibia | 16,000 |
| Tibia/Fibula (unilateral) | 20,000 |
| Tibia/Fibula (unknown sidedness) | 30,000 |
| Wrist | 20,000 |
| **CT Scans** | |
| Abdomen | 200,000 |
| Chest | 175,000 |
| Head | 175,000 |
| Pelvis | 175,000 |
| Spine | 262,500 |
| **MRIs** | |
| MRI scan* | 358,181·80 |
| **USSs** | |
| USS scan* | 35,935·48 |
| **Hospital admission and stay** | |
| General ward (daily) | 10,000 |
| ICU (daily) | 50,000 |
| **Death expenses** | |
| Death certificate | 15,000 |
| Corpse transport (from wards to morgue) | 15,000 |
| Body bag (plastic) | 50,000 |
| **average of all procedure types due to non-specificity in trauma registry record* | |
